# Uncovered Microbial Diversity in Antarctic Cryptoendolithic Communities Sampling Three Representative Locations of the Victoria Land

**DOI:** 10.3390/microorganisms8060942

**Published:** 2020-06-23

**Authors:** Claudia Coleine, Nuttapon Pombubpa, Laura Zucconi, Silvano Onofri, Benedetta Turchetti, Pietro Buzzini, Jason E. Stajich, Laura Selbmann

**Affiliations:** 1 Department of Ecological and Biological Sciences, University of Tuscia, 01100 Viterbo, Italy; zucconi@unitus.it (L.Z.); onofri@unitus.it (S.O.); 2 Department of Microbiology and Plant Pathology, University of California, Riverside, Riverside, CA 92521, USA; npomb001@ucr.edu (N.P.); jason.stajich@ucr.edu (J.E.S.); 3 Department of Agricultural, Food and Environmental Sciences, University of Perugia, 06121 Perugia, Italy; benedetta.turchetti@unipg.it (B.T.); pietro.buzzini@unipg.it (P.B.); 4 Italian National Antarctic Museum (MNA), Mycological Section, 16166 Genoa, Italy

**Keywords:** Antarctica, endolithic communities, habitat, sampling effort, fungi, bacteria, amplicon sequencing

## Abstract

The endolithic niche represents an ultimate refuge to microorganisms in the Mars-like environment of the Antarctic desert. In an era of rapid global change and desertification, the interest in these border ecosystems is increasing due to speculation on how they maintain balance and functionality at the dry limits of life. To assure a reliable estimation of microbial diversity, proper sampling must be planned in order to avoid the necessity of re-sampling as reaching these remote locations is risky and requires tremendous logistical and economical efforts. In this study, we seek to determine the minimum number of samples for uncovering comprehensive bacterial and fungal diversity, comparing communities in strict vicinity to each other. We selected three different locations of the Victoria Land (Continental Antarctica) at different altitudes and showing sandstone outcrops of a diverse nature and origin—Battleship promontory (834 m above sea level (a.s.l.), Southern VL), Trio Nunatak (1,470 m a.s.l., Northern VL) and Mt New Zealand (3,100 m a.s.l., Northern VL). Overall, we found that a wider sampling would be required to capture the whole amplitude of microbial diversity, particularly in Northern VL. We concluded that the inhomogeneity of the rock matrix and the stronger environmental pressure at higher altitudes may force the communities to a higher local diversification.

## 1. Introduction

Rocks represent an ancient terrestrial ecological niche, where microbes were the sole forms of life [1]. At present, microbial lithobionts still dominate environments where conditions prevent the settlement of less resistant and adapted organisms, eventually exploiting the endolithic airspaces when external environmental parameters become incompatible with active life. In fact, airspaces within rocks supply a shielded and buffered microenvironment for microbiota, providing protection from intense solar irradiation, desiccation, thermal fluctuations, and access to mineral nutrients [2]. Therefore, the ability to develop endolithically confers to specialized microbial communities the chance to thrive under different extreme conditions. Endolithic microorganisms have colonized many environments that border on the extreme limits of life, i.e., hot and cold deserts or geothermal environments [2,3,4,5] and all drylands worldwide [6]. These microbial communities play an important ecological role mediating inputs and outputs of gases, nutrients and water uptake from desert rock surfaces, regulating weathering, nutrient cycles and assuring the balance and functionality of these harshest ecosystems, creating positive feedback for further colonization and weathering [6,7]. Rock prokaryotic and eukaryotic microbial communities are, therefore, crucial in the preservation of drylands and a deep understanding of their diversity and functionality is of high importance in an era of global warming and rapid expansion of desertification.

Bare rocks dominate the landscape in the ice-free areas of Continental Antarctica and from the McMurdo Dry Valleys to mountain peaks rising from the Polar Plateau along the Victoria Land. They are the primary substratum for life, supporting the highest permanent biomass in these regions [8,9,10], described as the harshest cold and hyper-arid deserts on Earth. Despite different rock typologies, as dolerites and granites are present in this area, sandstone is by far the most widespread and suitable substratum for cryptoendoliths [11,12,13]. Among the typologies of cryptoendolithic colonization, the lichen-dominated community is the most complex and widespread [11]. These communities are self-supporting assemblages of phototrophic and heterotrophic microorganisms, including bacteria, Cyanobacteria, Chlorophyta and both free-living and lichen-forming fungi [4,14]. These are among the most stress-resistant organisms known to date, constantly living to the edge of their physiological adaptability [3,11].

Recent molecular studies are providing new insights into the distribution, biodiversity and composition of the Antarctic cryptoendolithic communities and a new understanding of their response to environmental pressure is arising [13,14,15,16,17,18,19]. Besides, these studies clearly highlighted that biodiversity is highly variable in rock samples, not only from different localities, but also for rocks coming right from the same locality [13]. The uncertainty in gathering the complete metacommunity hampers a deep understanding and an appropriate description of the biodiversity and functionality of these border ecosystems. Planning a proper sampling, assuring a reliable estimation of microbial biodiversity, is of importance for different reasons. Firstly, reaching these remote locations is risky and also requires tremendous logistical and economical efforts; therefore, each campaign should be well-planned to gain the highest results and avoid the necessity of successive re-sampling. Secondly, most of these locations are strictly protected, representing a nearly pristine environment, largely undisturbed and uncontaminated by humans. The McMurdo Dry Valleys, for instance, as a whole, are designated as ASMAs (Antarctic Specially Managed Areas), and include five different ASPAs (Antarctic Specially Protected Areas) to protect these outstanding environments. Specific permits are required to enter and the possibility of sampling is limited. Hence, the thorough definition of a proper sampling amplitude for biodiversity studies is critical to minimize the environmental impacts.

In a previous study, we made the first attempt to determine, with a metabarcoding approach, the minimum sampling effort of colonized rock samples collected along an area of about 100 m^2^ in Battleship Promontory, Southern Victoria Land (VL), Antarctica. Besides, the saturation curves did not reach the plateau, indicating that the number of sampling was not sufficient to gather the whole biodiversity in the rather wide area considered [20].

With the present study, we have sampled restricted locations, up to about 4 m^2^, to explore a homogeneous region to reduce sample variability. Moreover, three different habitats were sampled, representative of distinct environmental conditions in an altitudinal transect—Battleship promontory (BP, 834 m a.s.l.), Southern Victoria Land (SVL) and Trio Nunatak (TN, 1470 m a.s.l.), Mt New Zealand (NZ, 3100 m a.s.l.), and Northern Victoria Land (NVL). These habitats are characterized by the different nature of sandstone outcrops—in BP, the sedimentary rocks are typically a formation of the Beacon Supergroup, dating back to the Devonian–Triassic (400 to 250 MYA) and was composed mostly of orthoquartzite [21,22], while sandstones in the Northern Victoria Land have a more recent origin, dating from the Triassic to the Jurassic (252 to 145 MYA).

We focused on bacteria and fungi as the main components of these communities and we aimed to (i) test the effects of habitat constraints in the Antarctic endolithic settlement and highlight significant differences or overlapping in community structure and composition and (ii) determine whether eight samples would be enough to capture most bacterial and fungal species (at least 75%) in these communities. All the hypothesis included in the present study will give a more comprehensive knowledge of the amplitude and variability of microbial biodiversity in these edge ecosystems and will provide general recommendations for future experimental sampling design in the highly protected Antarctic desert, eventually applicable to drylands worldwide.

## 2. Materials and Methods

### 2.1. Study Area

**Figure 1:**
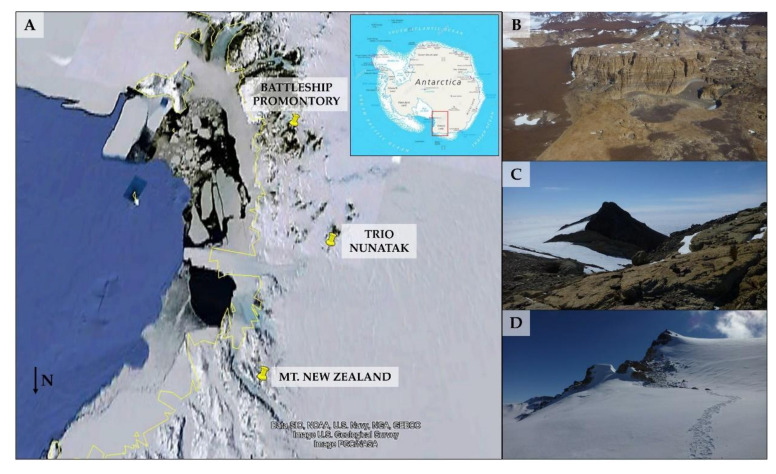
(**A**) Map of Antarctica—the Victoria Land (red square) is zoomed to show the sampling areas. The satellite photo was provided by Google Maps (2019 Google, TerraMetrics, Data SIO, NOAA, U.S.A. Navy, NGA, GEBCO). (**B**) Battleship Promontory; (**C**) Trio Nunatak; (**D**) Mt New Zealand.

Sandstones colonized by lichen-dominated cryptoendolithic communities were collected during the XXXI Italian Antarctic Expedition (December 2018–January 2019) at Battleship Promontory (BP; 76°54’04.0"S 160°54’36.6" E; 834 m a.s.l.; Beacon outcrops, Southern Victoria Land), Trio Nunatak (TN; 75°28’56.6"S 159°35’28.3" E; 1,470 m a.s.l.; Northern Victoria Land), and Mt New Zealand (NZ; 74°10’44.0"S 162°30’53.0" E; 3,100 m a.s.l.; Northern Victoria Land), as shown in Figure 1. Eight rock samples were aseptically collected from each site, respectively, within an area of about 4 m^2^, using a geological hammer and chisel. The presence of endolithic colonization was assessed by direct in situ observation through magnifying lenses. Samples were placed in sterile bags, preserved at −20 °C immediately upon collection to avoid modification and transported and stored at −20 °C at the University of Tuscia (Viterbo, Italy), until processed.

### 2.2. Metagenomics DNA Extraction and Amplicon Sequencing

Rocks were crushed in sterile conditions using a hammer and chisel; metagenomic DNA was extracted from 1 g of powdered rocks using a MOBIO Power Soil DNA Extraction kit (MOBIO Laboratories, Carlsbad, CA, USA).

The Ribosomal Internal Transcribed Sequence 1 region (ITS1) and V4 region of the 16S rRNA gene were targeted to assess the fungal and bacterial community membership, respectively. The ITS1 region was amplified using barcoded primers ITS1F/ITS2, developed for shorter read length [23] and V4 region using the new developed barcoded F515/R806 primer set as described by Caporaso and colleagues [24]. PCR was carried out with a total volume of 25 μL, containing 1 μL of each primer, 12.5 μL of Taq DNA Polymerase (Thermo Fisher Scientific Inc., Waltham, MA, USA), 9.5 μL of nuclease-free water (Sigma–Aldrich, St. Louis, MO, USA) and 5 ng of DNA template using an automated thermal cycler (BioRad, Hercules, CA, USA). The ITS1 locus was amplified following initial denaturation at 94 °C for 1 min, 35 cycles of denaturation at 94 °C for 30 s, annealing at 52 °C for 30 s, extension at 68 °C for 90 s, followed by a final extension at 68 °C for 7 min. The PCR for the V4 region followed a protocol of an initial denaturation at 94 °C for 3 min, 35 cycles of denaturation at 94 °C for 45 s, annealing at 50 °C for 1 min, extension at 72 °C for 90 s, followed by a final extension at 72 °C for 10 min. Amplicons, quantified by a Qubit dsDNA HS Assay Kit (Life Technologies, Carlsbad, CA, USA), were pooled and then purified with Qiagen PCR CleanUp kit (Macherey-Nagel, Hoerdt, France).

Paired-end sequencing (2 × 300 bp) was carried out on an Illumina MiSeq platform at the Institute for Integrative Genome Biology, University of California, Riverside, Riverside (CA) and at the Vincent J. Coates Genomics Sequencing Laboratory at University of California, Berkeley (CA).

### 2.3. Raw Data Processing

The ITS and 16S amplicon sequencing datasets were processed with AMPtk: Amplicon ToolKit for NGS data (formally UFITS) (https://github.com/nextgenusfs/amptk) v. 1.4.3 [25], removing barcodes and primers from raw data. Reads were then subjected to quality trimming to a maximum of 300 bp and discarding reads less than 100 bp in length, and chimera removal was performed utilizing USEARCH with default parameters v. 9.1.13 [26]. Sequence quality filtering was performed with the expected error parameter of 0.9 [27] and the cleaned dataset was clustered with UPARSE using a 97% percent identity parameter to generate the Operational Taxonomic Units (OTUs). Global singletons and rare taxa (<5 reads) were discarded as likely false positives due to sequencing errors, following Lindahl et al. [28]. Finally, taxonomic identification was performed with a hybrid database SINTAX/UTAX [26].

Raw sequencing data have been archived in the NCBI SRA database linked to BioProject accession numbers PRJNA453198 and PRJNA379160.

### 2.4. Accumulation and Extrapolation

Species accumulation curve analysis was implemented to estimate the expected number of new species given additional sampling effort [29,30]. The species accumulation curve is a graph of the expected number of detected species as a function of sampling efforts, representing the sampling process [31].

Curves were calculated using the PRIMER-E v. 7 implementing Plots > Species Accum Plot function. The increasing total number of different species observed (S) was plotted against samples successively pooled (Sobs—total number of species observed in all samples pooled). We used the ‘permute’ option to enter samples utilizing randomization procedures [32]. When the curves level off, and the number of OTUs does not increase by adding further samples, it indicates sufficient samples have been collected to accurately characterize the community [33].

We also computed an extrapolation model (sample-based extrapolation curve) with 95% unconditional confidence intervals, using the analytical formulas of Colwell et al. [34], for the samples that did not reach plateau. The model attempts to predict the asymptotic number of species that would be found for an increasing number of samples, estimating the number of samples needed to properly describe biodiversity, by using EstimateS v. 9 (Statistical Estimation of Species Richness and Shared Species from Samples).

For all tests, sample-based incidence data were used and analysis carried out with 999 permutations.

### 2.5. Downstream Analysis

Changes in community composition among the sampling sites were evaluated with nonmetric multidimensional scaling (NMDS) analysis, performed with PAST (v. 2.17) (PAleontological Statistics) [35] and their confidence intervals were investigated through permutational PERMANOVA *(p < 0.05)* [36]. We used Jaccard (incidence) and Bray–Curtis (relative abundance) metrics to calculate pairwise community distance matrices and examine differences in beta diversity. Abundance data were square-root transformed and analyses were carried out with 999 permutations.

Biodiversity indices such as Richness, Shannon’s diversity [37,38] (calculating using the natural log base e), and Simpson’s dominance [39] were calculated using Primer-E v. 7 software (PRIMER-E Ltd, Plymouth, UK). Differences in alpha diversity and taxonomic composition compared using one-way ANOVA with the “aov” function and pairwise multiple comparison (Tukey test) with the “Tukey HSD” function in R v. 3.5.1 using Phyloseq package [40]. A small probability *p*-value *(<0.05)* indicated a significant difference of community composition among all samples.

## 3. Results

### 3.1. Estimating and Determining Sampling Effort Across Sampling Areas

After preprocessing, a total of 2,141,716 fungal valid paired sequence reads were filtered, ranging from 13,115 to 243,988 per sample, while a total of 1,219,161 bacterial reads were generated across the samples, ranging from 10,867 to 119,961 reads per sample. The cleaned datasets, after quality trimming singletons and rare taxa removal (< 5 reads), were clustered using a 97% percent identity, generated 177 fungal OTUs, while a total of 449 bacterial OTUs were obtained (Supplementary Tables S1 and S2).

**Figure 2:**
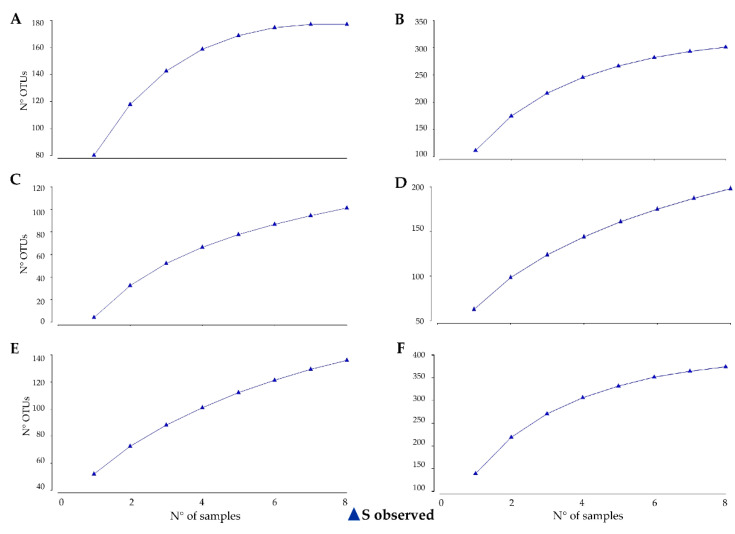
Fungal and bacterial species accumulation curves produced by the analytical formulae and randomizing the samples values were obtained using observed species (Sobs) counts and plotted against increasing numbers of samples. (**A**,**B**) Battleship Promontory, fungi and bacteria, respectively; (**C**,**D**) Trio Nunatak, fungi and bacteria, respectively; (**E**,**F**) Mt New Zealand, fungi and bacteria, respectively.

The fungal randomized accumulation curve reached saturation with our sampling effort and the asymptote indicated that 100% of the species (180) were detected when eight samples are collected only for BP (Figure 1A), while the curves did not reach a plateau, showing an increasing richness in the TN and NZ areas (Figure 2C,E). The total bacteria revealed were 300, almost 200 and more than 350 in BP, TN and NZ, respectively; nevertheless, the total bacterial diversity was not detected in all habitats, indicating that sampling was not exhaustive (Figure 2B,D,F).

An extrapolation analysis of empirical sample-based rarefaction curves was computed to estimate the number of species expected to be found in a larger number of samples from the same site. For fungi, the sampling required would be 23 and 28 samples for TN and NZ, respectively, showing that 150 and 190 fungal species may be present in these localities. A few more than 30 samples would be necessary for a comprehensive description of bacterial diversity of TN and NZ (for a total of 350 and 465 individuals, respectively), while 12 samples would be needed to capture all the putative 335 bacteria species occurring at BP (Table 1).

### 3.2. Differences in Community’ Composition by Site

To investigate the similarity of the communities’ composition amongst the three different sampling habitats, a nonmetric multidimensional scaling (NMDS) ordination was computed using both incidence and abundance data; since both approaches produced similar results, ordination plots based on abundance only were displayed.

**Figure 3:**
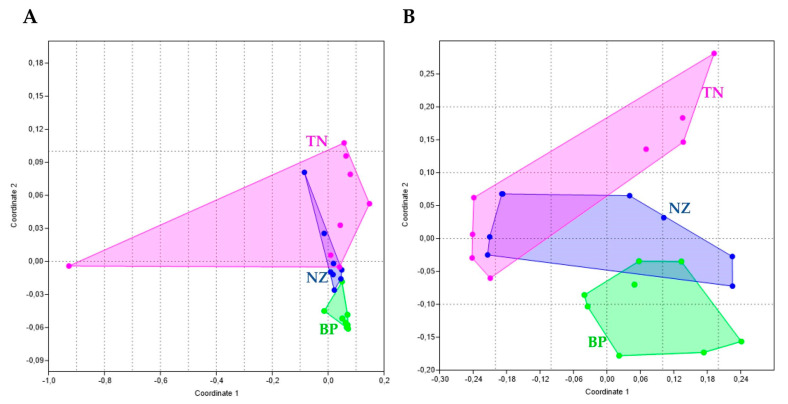
Nonmetric multidimensional scaling (NMDS) ordination plots, based on square-root transformed abundance data (PERMANOVA, *p* < 0.05). (**A**) Fungi, stress value = 0.11; (**B**) bacteria, stress value = 0.09.

Globally, the NMDS analysis revealed a strong structuring of both fungal and bacterial communities, showing the majority of samples clustered together by site according to PERMANOVA analysis (*p* < 0.05). The fungal community composition changed significantly between BP and TN, while a few samples collected at NZ clustered with BP and TN rocks (Figure 3A). Differences in bacterial beta diversity were visualized in Figure 3B, showing that prokaryotic communities clustered separately by sampling site.

**Figure 4:**
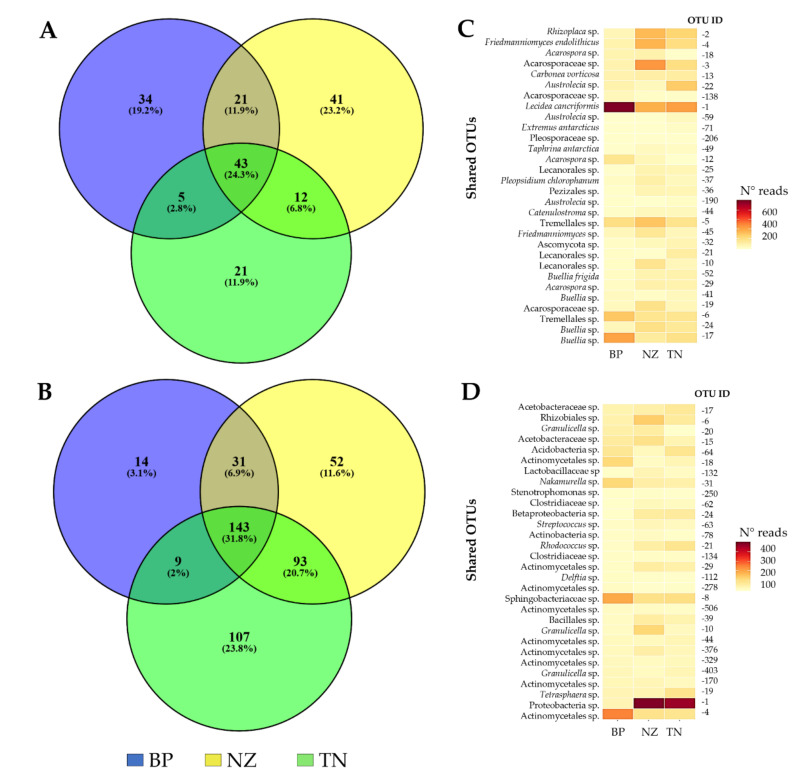
(**A**,**B**) Venn diagrams reporting the distribution of fungal (**A**) and bacterial (**B**) OTUs among the three localities studied. Percentages of both shared and unique OTUs are shown in parentheses. Heatmap of the most 30 representatives shared OTUs across all samples—(**C**), fungi; (**D**), bacteria.

Venn diagrams indicated that a substantial fraction of OTUs were shared among sites. For fungi, 23.4% (43 OTUs) were shared among the three different habitats (Figure 4A), including primarily lichens belonging to *Lecidea cancriformis* (e.g., OTU1), *Austrolecia* (OTU22), *Rhizoplaca* (OTU2), *Acarospora* (OTU3), *Buellia* (OTU17). A few microcolonial black fungi (i.e., *Friedmanniomyces endolithicus*, OTU4 and *Extremus antarcticus*, OTU71) and the endemic yeast *Taphrina antarctica* were also shared among the three habitats. The number of reads of the most 30 representative OTUs are mapped, showing a different relative abundance by site (Figure 4C); for instance, OTU1 was most abundant in BP, while the abundances of OTUs 2-3-4 were much higher at TN and NZ, while relatively absent at BP. Almost 20% was unique for BP and NZ (34 and 41 OTUs, respectively). The fungal community from TN exhibited the lowest percentage of unique taxa (11.9%, 21 taxa).

Among the three habitats, a great proportion of bacterial OTUs was found exclusively at TN (23.8%, 107 unique taxa), while BP showed the lowest number of unique taxa (14, 3.1%) and 52 OTUs (11.6%) were found at NZ. Conversely, the number of shared OTUs was higher in bacteria than in fungi; indeed, more than 30% of taxa were shared among the three groups, showing a different frequency across all habitats (Figure 4B,D), including members of Alphaproteobacteria (such as OTUs 15-6-17), Actinobacteria (e.g. OTUs 19 (*Tetrasphaera* sp.)-4-31-18-506), and Acidobacteria (OTUs 64-20-403).

### 3.3. Differences in Alpha Diversity and Taxonomic Composition

**Figure 5:**
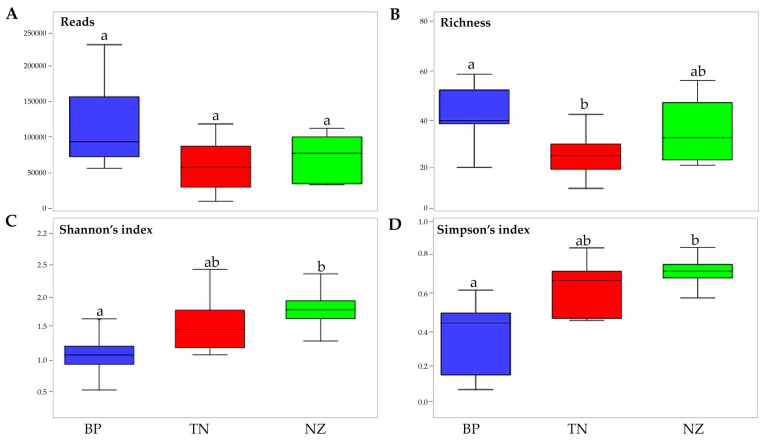
Fungal biodiversity indices. (**A**) Number of reads, (**B**) richness, (**C**) Shannon’s index, (**D**) Simpson’s index. Boxplots show 25th and 75th percentile, while error bars show 1st and 99th percentile. Same letters indicate that no significant differences occurred among the site, according to the one-way ANOVA Tukey HSD test (*p* < 0.05).

The relative abundance of the total fungal community exhibited no significant (*p* > 0.05) differences amongst the three sampling habitats (Figure 5A; Supplementary Table S3). On the contrary, richness in species among the three habitats showed the highest and lowest values (p < 0.05) in BP and TN, respectively; the biodiversity Shannon’s and Simpson’s dominance indices were significantly higher in NZ communities than for BP samples (*p* < 0.05) (Figure 5B–D; Supplementary Table S3).

When comparing bacterial communities, BP had a significantly lower relative abundance and species richness, while NZ exhibited higher values (*p* < 0.05) (Supplementary Figure S1A,B; Supplementary Table S3). Furthermore, we did not find significant changes in Shannon’s and Simpson’s indices according to habitat (Supplementary Figure S1C,D).

**Figure 6:**
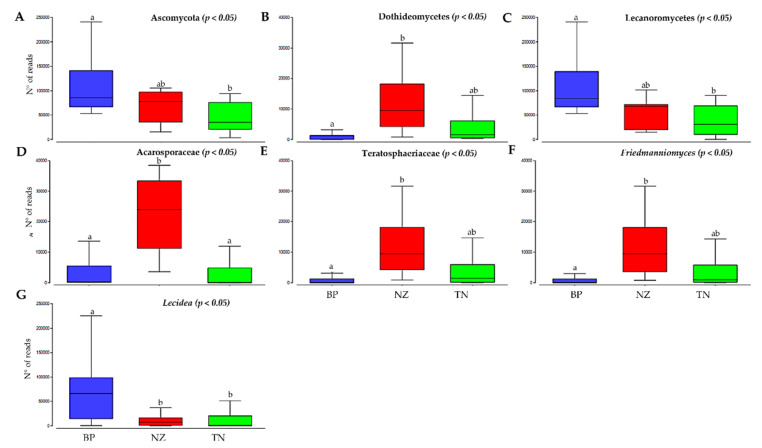
Relative abundance of Fungi. (**A**) Ascomycota phylum; (**B**) Dothideomycetes class; (**C**) Lecanoromycetes class; (**D**) Acarosporaceae family; (**E**) Teratosphaeriaceae family; (**F**) *Friedmanniomyces* genus; (**G**) *Lecidea* genus. Boxplots show 25th and 75th percentile, while error bars 1st and 99th percentile. Same letters indicate that no significant differences occur among site, according to the one-way ANOVA Tukey HSD test (*p* < 0.05).

We compared taxonomic composition for each microbial group by sampled sites. Significant differences in taxa abundances were found in only a few taxonomic groups. For fungi, three phyla, six classes, seven families and 12 genera have been observed across all samples (Supplementary Table S1). Fungal abundance comparison among sites showed significant differences within seven fungal classes (ANOVA, *p* < 0.05). In particular, we found that BP was enriched in members of the phylum Ascomycota, particularly in lichenized fungi, belonging to Lecanoromycetes and genus *Lecidea* (Figure 6A,C,G); the lichenized family Acarosporaceae was found to be enriched at NZ (Figure 6D). Besides, Dothideomycetes, Teratosphaeriaceae and the black fungal genus *Friedmanniomyces* abundances were at the greater abundance at the highest altitude at NZ (Figure 6B,E,F, Supplementary Table S4).

When bacterial communities were examined, we were able to retrieve six bacterial phyla and eight classes, while it was not possible to perform analysis at a higher taxonomic resolution as most of the OTUs were unidentified. Only two bacterial classes showed significant differences in abundance (ANOVA, *p* < 0.05)—Proteobacteria and Deinococci (Supplementary Figure S2, Supplementary Table S1). Specifically, both these groups showed a greater relative abundance at TN and NZ.

## 4. Discussion

To uncover the breadth of microbial diversity in Antarctic cryptoendolithic communities remains a challenging but essential step to deepen our understanding on these border ecosystems and on the microbes’ ability to exploit and be successful in the far extremes. A notable variability in biodiversity and structure in endolithic communities collected from the same location or in sites with similar environmental conditions have been reported by previous studies, e.g. [13,17]. A recent work sought to estimate how the sampling effort affected the accuracy of community diversity descriptions in a 100 m^2^ sampling area at Battleship Promontory, McMurdo Dry Valleys (Southern Victoria Land) [20].

In the present study, we investigated a selection of rocks collected in strict proximity to each other in three geographically different locations (along Northern and Southern Victoria Land) characterized by diverse sandstone typologies at increasing environmental pressure due to altitudinal increments (i.e., BP 1,000 to NZ 3,100 m a.s.l.).

The relative abundance obtained was invariably higher for fungi, up to three times greater than for bacteria. These data are consistent with results reported in previous studies highlighting the large predominance of fungi in these communities [20]. Despite this higher fungal abundance, the bacterial compartment was much more biodiverse, with an overall of 449 bacterial OTUs against 177 for fungi.

Overall, the curve of the species accumulation plots did not approach saturation, suggesting that additional sampling and sequencing would have recovered many other additional OTUs, with the exception of fungal communities collected at BP, the southernmost inspected site, the only one located in Southern Victoria Land. Indeed, fungal accumulation curves indicated that fungal diversity at BP was sufficiently captured with the current sampling depth and design, analyzing at least seven replicates, despite fungi being much more abundant in this location, compared with the other two. Conversely, a total of 23 and 28 samples are needed to reach a plateau at TN and NZ, respectively.

Besides, we captured only a subset of the total estimated bacterial diversity in all three habitats. Specifically, only four additional samples would be needed to describe total bacterial diversity in BP, for a total of 12 rock samples to collect and analyze where, based on the number of reads obtained, the bacterial compartment was less abundant; instead, more than 35 rocks are needed to capture all bacteria present at TN and NZ, where the number of reads obtained was more than double. Therefore, abundance and number of OTUs showed an opposite trend for fungi and bacteria in the three locations. Besides, both the microbial compartments gave an increased OTU estimation in the locations at NVL, particularly for the most stressed location, NZ. The sandstones of the Beacon Supergroup of McMurdo Dry Valleys, from which cryptoendolithic lichen communities were first discovered and described in 1982 [4], are well known for their extremely homogeneous texture and scarce matrices. Instead, the outcrops characterizing the Northern Victoria Land and particularly at Mt New Zealand are richer in matrices between the orthoquartzite grains. Moreover, at 3100 m a.s.l. (NZ), the environmental conditions are much harsher and it is plausible that even minimal microclimatic variations (e.g., humidity, temperature, wind) over a distance of few centimeters), even due to the topology of rock substratum, may have forced the communities to a higher local diversification, leading to higher variability observed. The high degree of variability may, therefore, not be related to the breadth of the sampling area, but rather linked to the homogeneity of the substratum and to the environmental stress; indeed, our study revealed that, even if sampling area is greatly reduced (4 m^2^ instead of 100 m^2^ of the previous sampling), further extensive sampling is required to identify the high variance in biodiversity, particularly in the NV Land.

Several factors have been identified that may putatively influence Antarctic cryptoendolithic distributions, both abiotic (e.g., sun exposure, altitude) [17,18,41] and biotic (fungi-bacteria interaction) [19]. In this study, the impact of habitat has been investigated. When samples are pooled by location, the NMDS analysis separated them based on the geography, showing that samples from one site were generally more similar to each other than those of the same type collected in different sites. Overall, this trend prevailed both for fungal and bacterial communities, showing significant differences by site. Besides, a few samples collected at NZ did not present a clear pattern in their fungal community composition, maybe due to the higher variability occurring there.

The observed difference between microbial communities can be attributed to their differences in environmental conditions due to habitat, including the structure of substrate; we, therefore, hypothesize that heterogeneity in the mineral composition of rock matrix may be a driving factor for microbial community composition in this ecosystem.

Venn diagrams revealed the overlap of the microbial OTUs across the three studied habitats, reporting a diverse fraction unique to each habitat and represented by rarer, habitat-specific taxa. Indeed, in fungi, more than 50% of OTUs were identified as a unique fraction (i.e., only detected in one habitat), suggesting that these specialist fungal taxa show strong habitat preferences. In particular, we found 23% OTUs specific for the harshest habitat (NZ), while BP and TN showed lower percentages of unique taxa (19.2% and 11.9%, respectively) suggesting that the increasing environmental pressure may have led to the selection of hyperadapted species at the highest altitude. A similar trend has been observed analyzing the bacterial counterpart. The three sampled habitats sustain distinct specialist microbial taxa, as the majority of OTUs are found exclusively in one or the other communities; differently from fungi, TN possessed the highest unique OTUs (23.8%), while only 3% and 11% of OTUs were exclusively observed at BP and NZ, respectively, suggesting that bacteria do not have the same stress ability of fungi, and, at the highest altitude, fewer peculiar species are selected. Due to the fluctuating environmental conditions (e.g. any perturbation due to climate change), habitat specialists might be more vulnerable and, therefore, more prone to become extinct compared to habitat generalists.

Yet, more than 20% of generalist fungal taxa were shared across all sites, mainly lichens and black fungi, but also a few yeasts such as *T. antarctica* that were present in all three habitats, confirming the ubiquitous nature of this species in the Antarctic environments [42].

Bacterial communities showed a larger fraction of generalist taxa (more than 30%), among which members of Alphaproteobacteria were predominant. These data indicated that shared taxa represent the core of these communities and might play a critical role for the maintenance of cryptoendolithic ecosystem biodiversity and functioning.

Changes in bacterial and fungal communities were also overall highlighted when biodiversity indices have been considered. The lowest value of fungi richness was found at TN, while bacterial richness was higher at TN and NZ. We reported no difference in bacterial diversity among the habitats; conversely, interestingly, shifts in fungal community structure were observed between BP and NZ, where the latter showed highest values for Shannon’s and Simpson’s indices. A possible explanation suggests that due to more severe external conditions, endolithic communities inhabiting NZ are more adapted and dominated by a relatively small number of microbial taxa but that harbor a wide array of rare, yet more diverse microorganisms.

Bacterial species richness comparison by site indicated that most groups were not different by geography, while the distribution of abundance varied among a few bacterial phyla. The distribution of common phyla shows a dramatic increase in the percentage of reads attributed to Deinococci with increasing environmental pressure (from BP to NZ). There is also an increment, albeit less pronounced, in the Proteobacteria phylum. Previous studies found Proteobacteria to be a dominant component of other cold-extreme habitats including Antarctic soil biotopes [43,44], cryoconite holes [45] and cryptoendolithic communities both from the Arctic and Antarctic [46,47,48], suggesting that these heterotrophic bacteria are critical for functioning ecosystems of Polar regions. Yet, *Deinococcus*-like organisms such as *D. radiodurans*, *D. geothermalis* and *D. murrayi* are extremely radio-resistant species, well-known for their ability to withstand the high solar irradiation of the South Pole, especially large amounts of UV, and also to survive to ionizing radiation, limiting damage to their DNA [49,50,51].

Among fungi, we found that the lichen-forming fungi belonging to Lecanoromycetes (Dothideomycetes), and in particular members of *Lecidea* genus, were most abundant at BP, where conditions are slightly more permissive. In contrast, we reported an increase in abundance of black fungi belonging to Teratosphaeriaceae (Capnodiales, Dothideomycetes) in the harshest conditions of NZ. Overall, the distinct distribution of these fungi among the different locations indicated a notable diverse preference in these microorganisms. Black fungi are known for their success under stressful conditions (e.g. high solar irradiation, drought, extreme temperature shifts), characterizing also the Antarctic deserts. Indeed, black fungi are recurrent colonizers of extreme habitats, from hot and cold deserts, rock surfaces and glaciers, saltpans, and acidic or polluted environments [52,53,54,55,56,57]. The increasing dominance of this group of fungi in the face of increasing pressure was also observed in the south-exposed Antarctic shaded rocks which are prone to stronger environmental constraints [18]; this feature makes black fungi particularly suitable to define the limit of extinction in the Mars-like Antarctic deserts.

In particular, the endemic black fungus *F. endolithicus* represents the most widespread and frequently isolated from Antarctic endolithic communities, up to 3300 m a.s.l. [17,41], suggesting a high degree of adaptation and specialization to the prohibitive conditions of such environments. Recently, the whole genome of *F. endolithicus* CCFEE 5311 was sequenced and the genome size was 46.75 Mbp; genomic traits in response to salt, X-rays, cold and DNA damage stresses have been identified, confirming exceptional poly-extremo tolerance of this species to survive across a wide variety of stresses [58].

## 5. Conclusions

Starting from the results of previous investigations, evidently showing the high variability of biodiversity in Antarctic cryptoendolithic communities among different samples, we based the present study on rock surveys in three locations along the Victoria Land (Continental Antarctica). In each location, eight different samples have been collected in a restricted area to emphasize the importance of sampling efforts for a comprehensive biodiversity description. We found clear indications that the increasing environmental pressure at highest altitudes, coupled with the inhomogeneity of the rock matrix characterizing Trio Nunatak and Mt New Zealand (Northern Victoria Land), forced the community to a higher local diversification; therefore, these locations require a much wider sampling for a proper biodiversity description. Moreover, our data suggest that the structure and chemical composition of rock matrix has a strong selective effect on the endolithic community settlement and composition.

Whilst we found that samples ranging from six to ten specimens would be sufficient to capture the whole microbial diversity in the McMurdo Dry Valleys, for the other localities the accumulation curves did not reach the plateau, indicating that it would be very difficult, if not impossible, to cover the complete biodiversity in all cases, unless analyzing a huge number of samples. Besides, since we were able to capture at least the 75% of both fungal and bacterial diversity in all the conditions, we can assert that the application of this protocol in future investigations may assure that the uncovered biodiversity would never exceed 25%. Further studies are needed to confirm similar trends in other locations in the Victoria Land and to characterize other microbial compartments of Antarctic endoliths (namely archaea and algae).

This work also provides the first detailed comparison of Antarctic rock-inhabiting communities in different habitats, offering new insights into the geographic patterns of these microbial ecosystems. Similar investigations may be applied in drylands worldwide, still largely unknown. In an era of global warming and rapid desertification, the study of these self-supporting microbial communities dwelling inside rocks in arid areas is of utmost importance since they play a pivotal role in the balance and functioning of these ultimate ecosystems, regulating water retention and nutrient cycles and creating positive feedback for further colonization.

## Figures and Tables

**Table 1:** Expected species richness values were computed for increased reference samples (sample-based extrapolation curves) using EstimateS with 95% confidence intervals.

	Site	Samples	N° of Species (Computed)
**Fungi**			
	Trio Nunatak	23	150
	Mt New Zealand	28	190
**Bacteria**			
	Battleship Promontory	12	335
	Trio Nunatak	32	350
	Mt New Zealand	34	465

## Supplementary Materials

The following are available online at https://www.mdpi.com/2076-2607/8/6/942/s1; **Figure S1.** Bacterial biodiversity indices. (**A**) Number of reads, (**B**) richness, (**C**) Shannon’s index, (**D**) Simpson’s index. Boxplots show 25th and 75th percentile, while error bars show 1st and 99th percentile. Same letters indicate no significant differences in one-way ANOVA Tukey HSD test (*p* < 0.05); **Figure S2.** Relative abundance of bacteria. (**A**) Proteobacteria class; (**B**) Deinococci Class. Boxplots show 25th and 75th percentile, while error bars 1st and 99th. percentile. Tukey HSD significant differences (*p* < 0.05) are indicated by different letters.; **Table S1**. Fungal taxonomy results at 97% of identity. Taxonomy assignment was performed with SINTAX/UTAX databases (Edgar, 2010); **Table S2.** Bacterial taxonomy results at 97% of identity. Taxonomy assignment was performed with SINTAX/UTAX databases (Edgar, 2010); **Table S3.** One-way ANOVA, F-value and Tukey HSD outputs both for fungi and bacteria; **Table S4.** One-way ANOVA, F-value and Tukey HSD outputs for alfa diversity comparison for fungi and bacteria, respectively.
