# Supplementary figures and images for: Uncovered Microbial Diversity in Antarctic Cryptoendolithic Communities Sampling Three Representative Locations of the Victoria Land
Source: Microorganisms. 2020 Jun 23;8(6):942. doi: 10.3390/microorganisms8060942 (PMC7356261; doi:10.3390/microorganisms8060942)

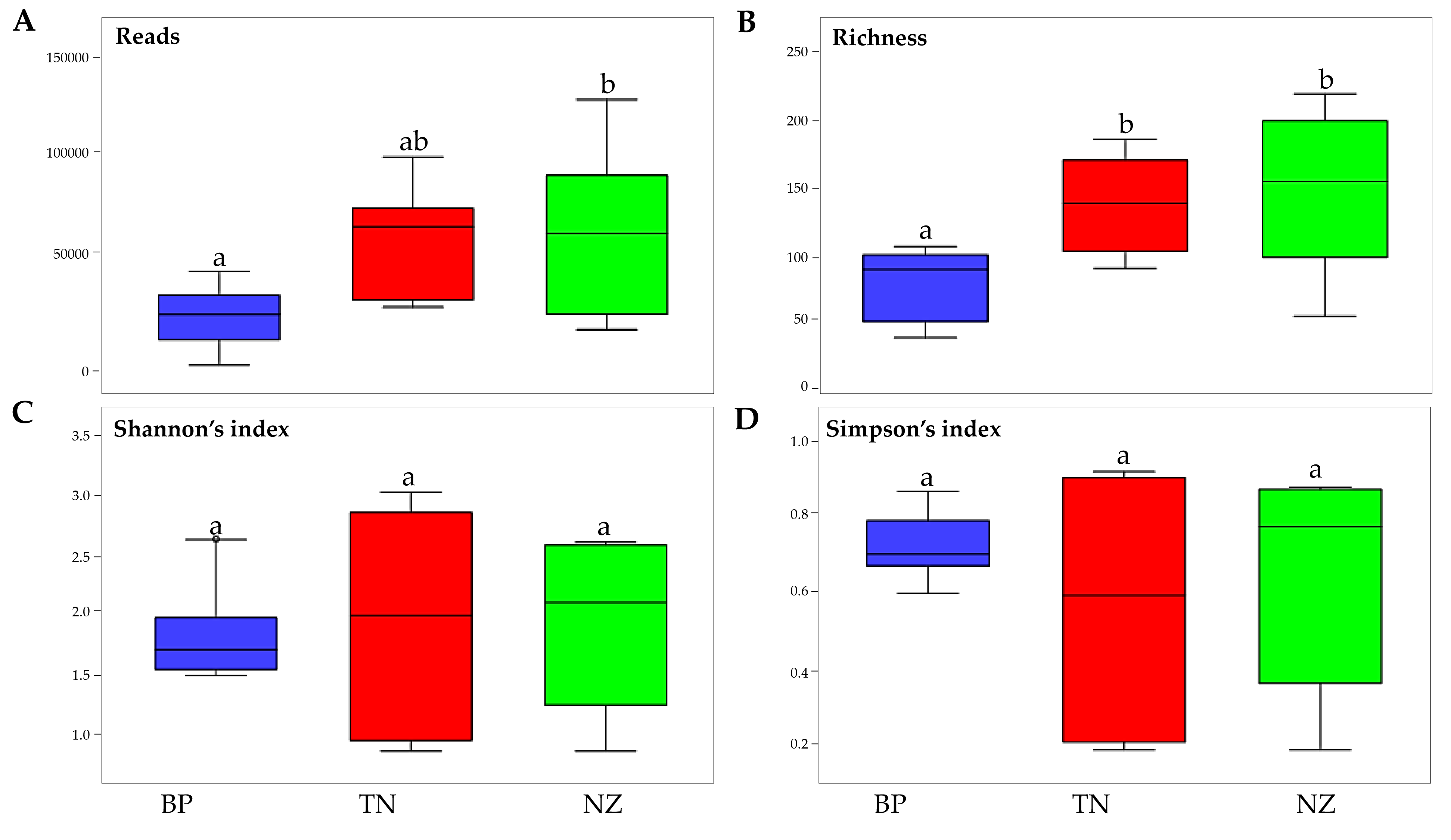

Supplement: Supplementary file 1 [file microorganisms-08-00942-s001.zip › Figure S1.tif]

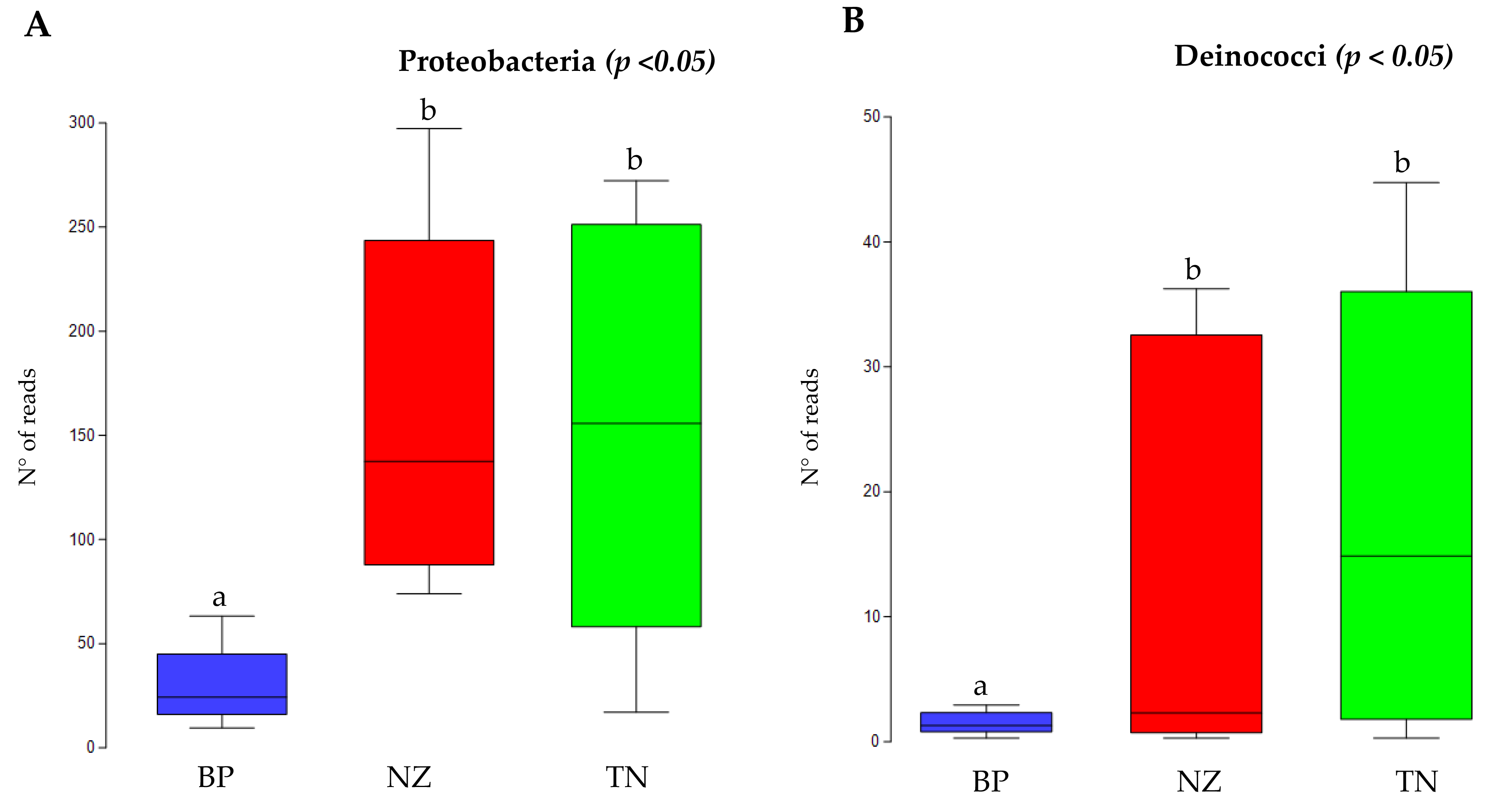

Supplement: Supplementary file 1 [file microorganisms-08-00942-s001.zip › Figure S2.tif]
